# A performance-based evaluation of chemically similar (carbonate) tempers from Late Prehistoric (AD 1200-1700) Ohio: Implications for human selection and production of ceramic technology

**DOI:** 10.1371/journal.pone.0194992

**Published:** 2018-03-26

**Authors:** Michelle Rae Bebber, Linda B. Spurlock, Michael Fisch

**Affiliations:** 1 Department of Anthropology, Kent State University, Kent, OH, United States of America; 2 College of Aeronautics and Engineering, Kent State University, Kent, OH, United States of America; Seoul National University College of Medicine, REPUBLIC OF KOREA

## Abstract

**Purpose:**

This study was designed to assess the mechanical properties of two calcium carbonate tempers, limestone and burnt shell. These tempers have been previously compared, in separate studies, to silicate-based grit or sand temper and, relative to the latter, are assumed to possess similar mechanical properties. However, their simultaneous use at the Morrison Village site begs the question: do these two calcium carbonate tempers indeed possess similar mechanical properties? In order to assess their performance characteristics, a side-by-side controlled experimental test was conducted to determine the degree of similarity in providing increased vessel strength and toughness.

**Methods:**

Standardized ceramic test samples were systematically prepared via a set, explicit protocol. An Instron Series IX universal testing machine configured with a four-point flexural test jig was used to perform a flexural strength test of the test samples. The Instron load and deflection data were used to calculate three values related to mechanical performance: *peak load*, *modulus of rupture*, and *modulus of elasticity*.

**Results:**

All four comparative tests clearly show substantial differences in *peak load*, *modulus of rupture*, and *modulus of elasticity*. These differences are statistically significant for each performance attribute in every iteration of the experiment and as indicated by Mann-Whitney *U* Tests.

**Conclusions:**

These results do not support the hypothesis that limestone and burnt shell offer the same performance characteristics. These results have implications for our understanding of prehistoric human selection of temper and the evolution of ceramic technology. Although both carbonate-based tempers are currently thought to offer the same benefits during the initial phase of pottery production, their contrasting post firing properties would have provided distinct benefits in different contexts. Future assessments of the Morrison Village ceramic assemblage should focus on residue analysis, or other functional indicators, to support or falsify this hypothesis.

## Introduction

A recurring question in studies of ceramic technology is whether or not pottery additives (i.e. tempers) were selected by prehistoric people for *production-based* benefits facilitating initial vessel formation, or for *performance-based* benefits associated with post-firing vessel use, and the precise nature of these production and/or performance benefits [1, 2, 3]. Many authors propose that temper was added to clay to assist in vessel formation [4, 5, 6, 7], whereas other studies have focused more on the variation in post firing mechanical properties between various temper types [2, 3, 8, 9, 10, 11, 12, 13, 14]. These studies have conclusively demonstrated that temper in general does provide benefits to both vessel formation and post firing vessel performance. However, more experimental work is needed to determine whether, and precisely what kinds of, production-based and/or performance-based functions were likely the motivating factor(s) driving particular types of temper adoption, use, and subsequent change over time. The present study addresses this question by conducting an archaeological experiment [15, 16, 17] comparing two carbonate-based tempers: limestone and burnt shell. It has been suggested that both calcium carbonate-based tempers function in a similar manner during the initial phase of vessel formation, essentially making clay more workable via calcium ion transfer [9, 18]. However, less is known about their comparative post firing performance attributes, and whether there are differential effects on overall vessel use-life shown by each of the carbonate tempers.

Benefits associated with tempers high in calcium carbonate (e.g., limestone, shell, or calcareous sand)–relative to non-calcium carbonate tempers (e.g. silicate-based grit or sand)–relate to both pre-fired raw material workability and post firing vessel performance. Previous studies of calcium carbonate-based tempers [9] have shown that they provide a selective advantage due to the flocculation that takes place as the calcium ions interact with the moist clay. This process causes the clay body to become more workable, which in turn facilitates the creation of larger, thinner vessels.

**Fig 1:**
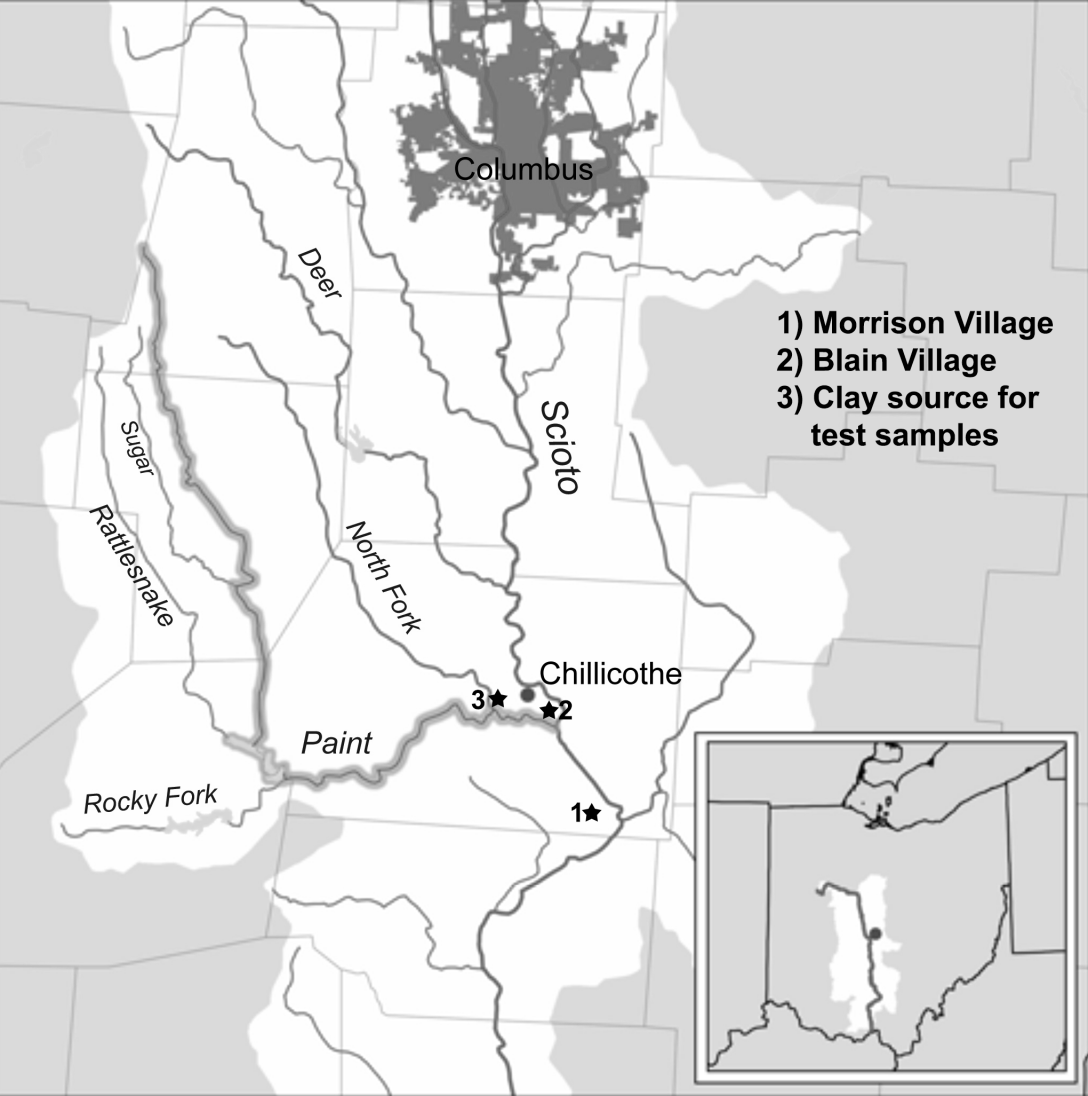
Map of Scioto River Valley, Ohio. Location of 1) Morrison Village site, 2) Blain Village Site, and 3) location of clay used for experimental sample production.

The temporal evolution of these production advantages are evident at Late Prehistoric archaeological sites in Southern Ohio (Fig 1) as temper selection shifted from silicate-based grit temper to carbonate-based tempers of either limestone or burnt shell, even though pottery manufacturing techniques and decorative styles remained relatively consistent [19, 20]. For example, at the Late Prehistoric site Morrison Village, the thickness of grit tempered sherds ranged from 8–11 mm, whereas the thickness range of limestone tempered sherds was 5–10 mm, and shell tempered sherds ranged from 5–8 mm thick [20]. Likewise, at the Late Prehistoric site Blain Village, the thickness of grit tempered sherds ranged from 3–20 mm with an average of 7.8 mm, whereas the shell tempered variety ranged from 3–12 mm with an average thickness of 6.3 mm [21].

In a previous study [9], researchers used an experimental approach to address the sudden appearance of limestone temper in the Late Woodland Period of Missouri (AD 400–900). Although the limestone tempered samples exhibited low deformation capacity—which represents low overall toughness—they [9] conclude that due to the reduced thermal gradient, limestone temper would provide better thermal shock resistance. This is based on the assertion that thinner vessel walls are more resistant to thermal shock [6, 9]. However, although limestone tempered vessels exhibit an increase in initial fracture resistance, once they reach peak load, they fail abruptly. This is in contrast to the post-peak response of (burnt) shell tempered pottery. Previous research [10, 11] indicates that the superior fracture toughness exhibited by shell temper is more so a result of its plate-like physical microstructure than its chemical structure. The deflection of microcracks around the plate-like microstructure of shell temper functions to dissipate energy more readily than the block-like physical microstructure of limestone particles, which increase the post-peak toughness value [9].

**Fig 2:**
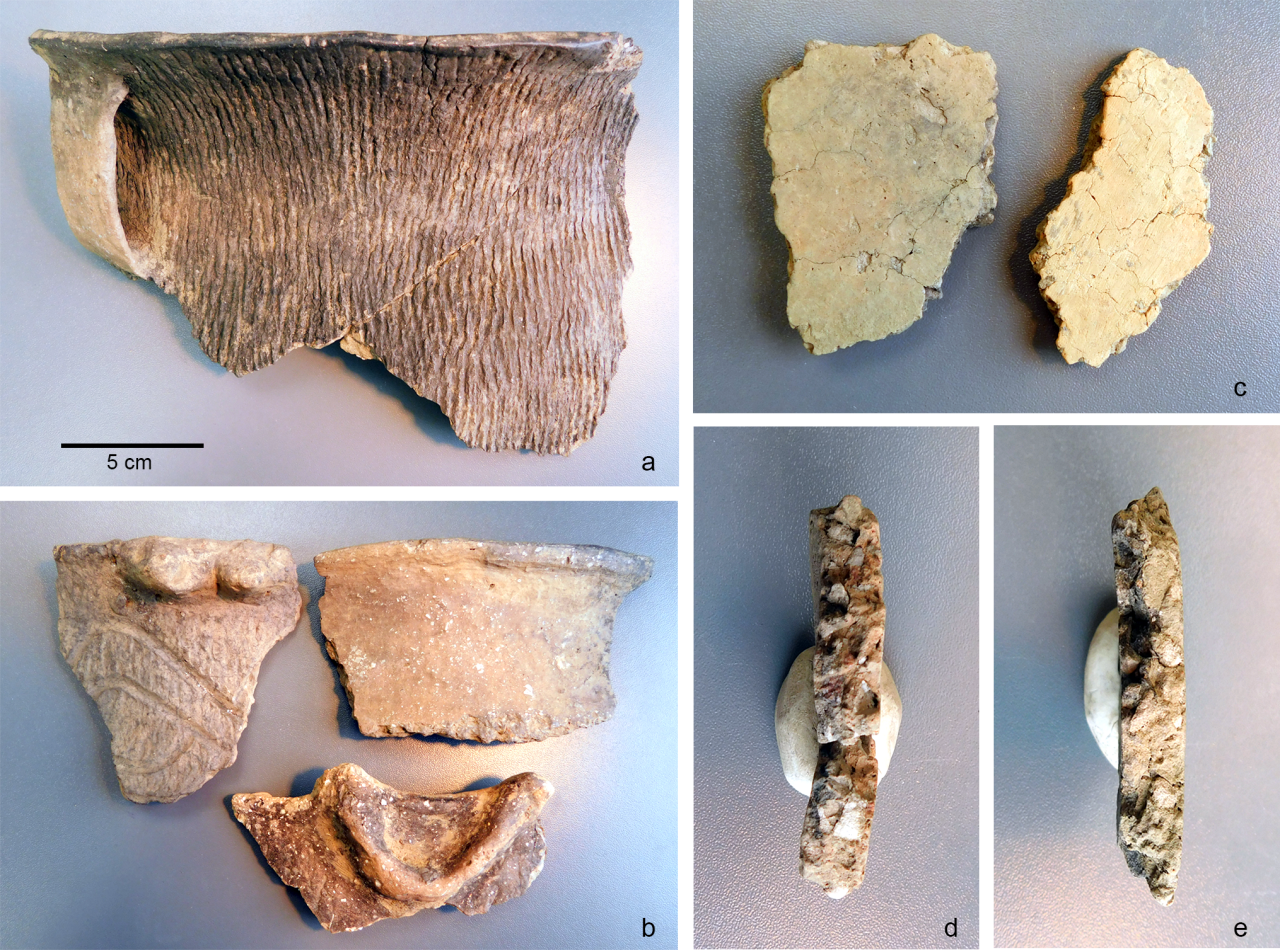
Pottery samples: a) Morrison Village shell tempered, b) Blain Village shell tempered, c-e) Morrison Village limestone tempered.

If these seemingly contrasting results from these two distinct studies [9, 10] can be confirmed, then it would mean that although chemically similar, limestone and burnt shell do not actually offer the same performance characteristics. Although limestone and shell have been tested independently as tempers [8, 9, 10, 11, 12, 14], they have never been directly tested side-by-side to fully evaluate their respective characteristics. The ceramic assemblage from the Late Prehistoric site of Morrison Village (c. AD 1700) provides an opportunity to directly apply such a test (Fig 2). Here, both limestone-tempered and shell-tempered vessels are found in close association. The pottery type *Morrison Plain* was defined based on the assemblage (n = 427 sherds) from the Morrison Village site. This type is considered to be a descendent of the Late Woodland Scioto Tradition and represents the latest prehistoric occupation in the Scioto River Valley immediately predating European contact [20]. The *Morrison Plain* type is defined as a smooth surfaced variety featuring large pieces of limestone temper. Additionally, three types of Late Prehistoric shell tempered, cordmarked pottery are also found at the Morrison Village site [20].

Of interest to the current study is the mechanical properties of these two calcium carbonate tempers. They have been previously compared separately to silicate-based grit or sand temper [9, 10, 11, 12], and, relative to the latter, are assumed to possess similar mechanical properties. However, their simultaneous use at Morrison Village begs the question: do these two calcium carbonate tempers indeed possess similar mechanical properties? If they provide the same benefits to post firing vessel performance, they could have been used interchangeably. Thus, a “supply-side” argument can be made: perhaps not enough of each temper type was available, or could be procured, for vessel production, therefore requiring the use of both carbonate-based tempers at Morrison Village. Alternatively, if limestone and burnt shell tempers yield different post firing mechanical properties, then this indicates that the carbonate tempers were not being selected for their effects on overall vessel use life. Thus, a production-based argument, relating to the manufacturing sequence, for their simultaneous selection can be made. As such, a direct, side-by-side controlled comparative experimental test of these tempers is warranted to determine the degree of similarity in providing increased vessel strength and toughness.

## Materials and methods

Cochrane [22] suggests an experimental design using “character suites” to identify possible relationships between multiple factors involved in pottery design. The current study follows this suggestion by creating 4 sample sets grouped by two (2) temper types, two (2) firing temperatures, and two (2) sample thickness, to determine if various types of temper may be providing functions that are unique to or possibly interrelated with these other factors.

### Sample description

For this study, it was determined that locally sourced clay and tempers would provide the best possible “realistic” assessment (i.e. high external validity) [23, 24] of the interactions between a naturally occurring clay and locally derived temper that could have been used during Late Prehistoric pottery manufacture of the Midwestern U.S.A. in general, and South-Central Ohio in particular.

Full details of clay and temper sourcing, processing, and ceramic test sample manufacture, are provided elsewhere [1], but a summary is provided here. In order to create ceramic test samples that accurately reflected sherds from the archaeological record, macroscopic and microscopic analyses of the clay matrix and temper used in pottery samples ranging from Early Woodland (800–100 B.C.) through Late Prehistoric contexts (AD 900–1400) were conducted. Thin-section petrographic analysis was also conducted to accurately characterize data regarding temper found in the archaeological samples. Mineralogical analysis via optical petrography was used to generate both qualitative and quantitative data. The petrographic data were used to create a mineralogical profile of key temper characteristics of size, density, orientation, and type.

The clay used in the present study was sourced in its raw form from the Scioto River Valley (Fig 1). The exact location of the clay deposit (39° 21' 12.6" N, 83° 03' 14.4" W) is along North Paint Creek, a major tributary to the Scioto River. Permission was granted by the property owner to access the area and remove the clay. The clay was removed from the riverbank, loaded into buckets by hand and shovel, and brought to the lab for processing. X-ray diffraction analysis, performed in order to identify the type of clay minerals present in the raw material, showed that the predominant mineral is illite [25, 26]. Quartz, dolomite, ankerite, and iron minerals are also present in the clay sample in low concentrations. With respect to tempers, limestone was locally procured from Ohio, while the fresh water mussel shell was sourced from the shores of the Scioto and Muskingum Rivers, where native populations of mussel are abundant.

Standardized ceramic test samples (Fig 2) were systematically prepared via a set, explicit protocol. (1) Two sample thicknesses (7 mm and 14 mm) were decided upon after consulting archaeological data from the sites sampled. As such, the final size of the experimental test samples after firing was 7T x 14W x 100L mm for the thinner set and 14T x 14W x 100L mm for the thicker set. Two temperatures, representing the minimum and maximum range likely to occur in an open firing, were selected for firing the samples [27, 28]. The lower firing temperature was 560°C, which is near the average temperature for low-fired pottery [29]. The higher firing temperature was 760°C in order to evaluate the upper range of “open” firing temperature on the selected tempers. Ethnoarchaeological data from open firings in many different cultures around the world support this generally high temperature range for open “bonfire” style firing [27, 28].

The samples were fired in a Skutt electric kiln to ensure precise temperature control and to avoid the uneven firing that often occurs in open “bonfire” regimes. Samples were heated 33°C per hour up to 99°C and then held at 99°C for eight hours to drive off any remaining water and to prevent steam build up which would ultimately cause failure. The samples were then heated at 50°C per hour until 300°C, and then heated at 100°C per hour until the maximum temperature of either 560°C or 760°C was attained. The samples were held at this temperature for 30 minutes to complete the firing cycle, and then left in the kiln to cool [9, 10].

After firing, the samples were examined macroscopically to assess temper particle orientation. Of primary interest was the orientation of the shell temper particles. It was observed that the shell temper in the samples showed preferred platy orientation in a manner akin to that of the archaeological samples. This orientation occurred as a result of the paddling into the mold during formation. Limestone particles were distributed evenly throughout the sample matrix.

### Experimental mechanical testing

**Fig 3:**
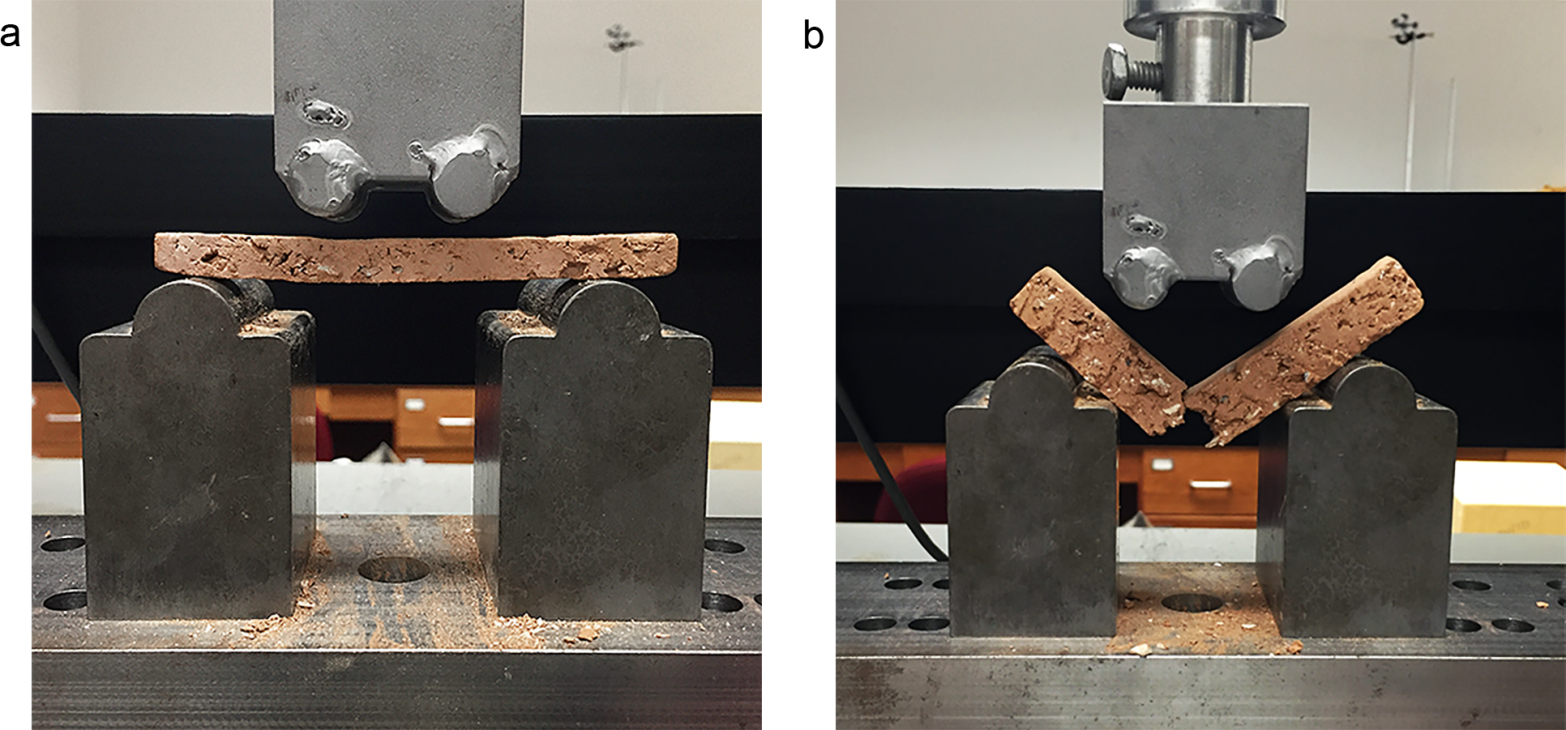
Instron 4-point bend test. Apparatus showing shell tempered samples (thin on left, thick on right).

An Instron Series IX universal testing machine configured with a four-point flexural test jig was used to perform a flexural strength test (Fig 3A). This type of test continuously measures applied load and the associated deflection. In this study, the stress was introduced and controlled entirely by the Instron testing apparatus. A crosshead displacement rate of 1.27 mm/per minute was used with a lower span of 7.62 cm. The test involves placing the pre-measured sample onto the testing apparatus lower crossbar. The balance is then reset to zero and the load bearing upper section begins to lower until contact is made with the sample. The load is continuously measured as the sample is deformed as a result of the applied force (Fig 3B). The Instron Series IX software provided with the testing apparatus continuously collected measurements throughout the test duration. The Instron data were used to generate deflection curves using Microsoft Excel Version 15.26.

The relationships between these parameters were used to assess the samples’ strength and overall toughness. The Instron load and deflection data were used to calculate three values related to mechanical performance: *peak load*, *modulus of rupture*, and *modulus of elasticity*.

*Peak load* refers to the maximum load applied during the test before the sample cracks. This measure provides an assessment of initial strength prior to fracture by measuring the maximum of amount load withstood by the sample before crack initiation.

*Modulus of rupture* is a value that denotes flexural strength. It is a measure of the amount of stress at fracture [30]. The modulus of rupture measure also provides an assessment of initial strength prior to fracture. Modulus of rupture is also referred to as bend strength or fracture strength.

*Modulus of elasticity* is a measure of a material’s ability to resist longitudinal deformation while under stress from an applied force [30]. The modulus of elasticity is calculated using the relationship between the maximum force at breaking, sample geometry, and the maximum deflection at breaking.

### Statistical analyses

Data were analyzed using SPSS statistical software version 23. To assess differences between the limestone tempered and shell tempered sets, we used nonparametric Mann-Whitney *U* tests. This is a conservative statistical procedure that requires only minimal assumptions of the data [31, 32]. Here, we report *p* values based on exact significance [BONFERONNI]. Four tests comparing limestone versus shell were undertaken in each of the three mechanical performance attributes (i.e. *peak load*, *modulus of rupture*, and *modulus of elasticity*) based on both thickness and firing temperature: 1) Thick (14 mm) samples fired to 560°C; 2) Thick (14 mm) samples fired to 760°C; 3) Thin (7 mm) samples fired to 560°C; 4) Thin (7 mm) samples fired to 760°C. The dependent variables of *peak load*, *modulus of rupture*, and *modulus of elasticity* were independently analyzed.

## Results

**Fig 4:**
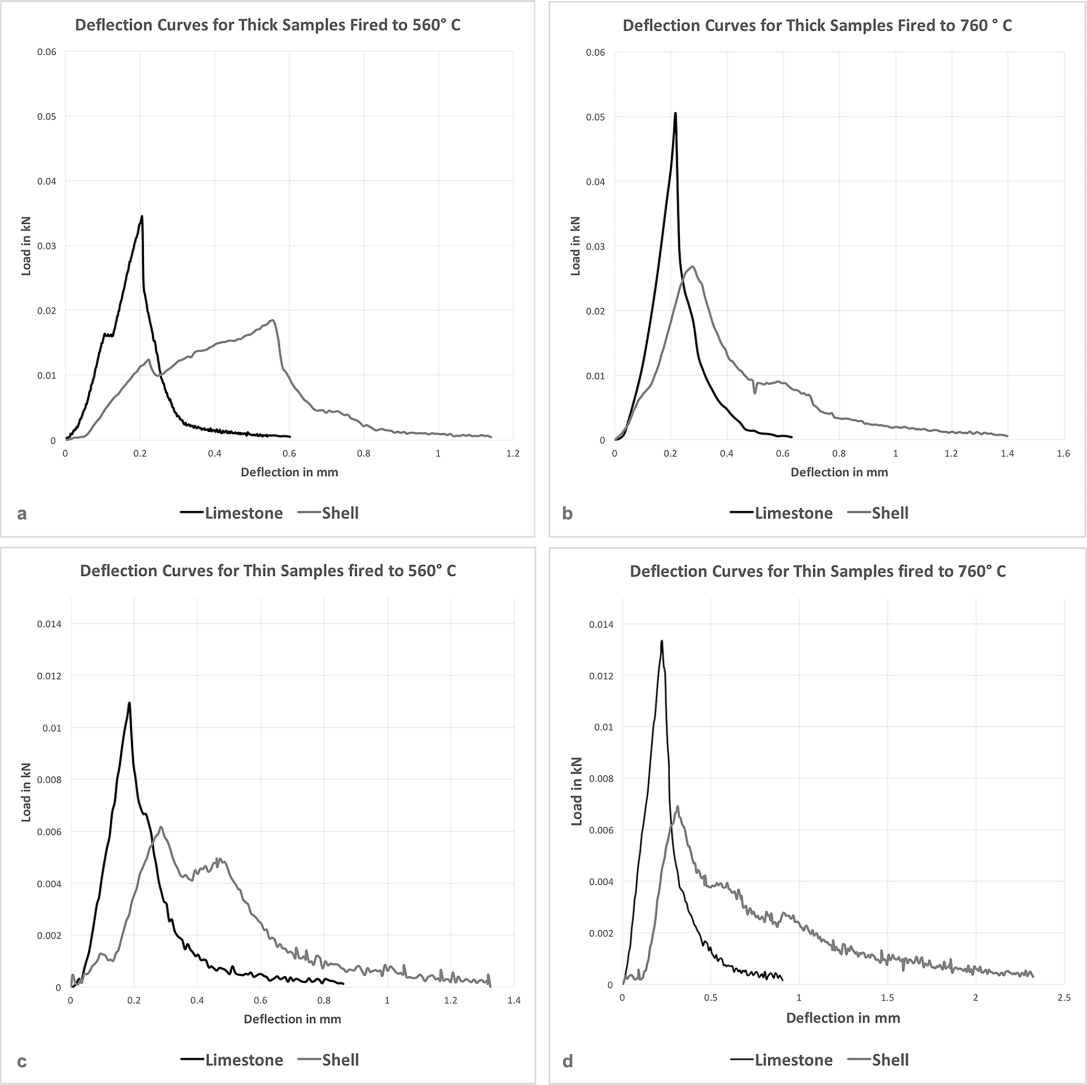
Deflection curve comparisons. Charts showing relationships between tempers: grit, limestone, and burnt shell.

Deflection curves are shown in Fig 4. All four comparative tests clearly show substantial differences in *peak load*, *modulus of rupture*, and *modulus of elasticity*. These graphical differences are also statistically significant for each performance attribute in every iteration of the experiment and as indicated by Mann-Whitney *U* Tests (Table 1). These results do not support the hypothesis that limestone and burnt shell offer the same performance characteristics.

**Table 1:** Statistical results of nonparametric Mann-Whitney *U* tests.

Test								
560, Thin	Variable	Limestone N	Limestone Mean	Shell N	Shell Mean	*U*	z	*p*
	Peak Load	10	0.010814	7	0.00509375	6.000	-2.943	0.002
	Modulus of Rupture		1145204.105		601965.7207	5.000	-3.034	0.001
	Modulus of Elasticity		1761333899		964246587	15.000	-2.128	0.035
								
560, Thick	Peak Load	9	0.033094444	12	0.01759	4.000	-3.553	<0.001
	Modulus of Rupture		1251591.613		652175.8737	7.000	-3.340	<0.001
	Modulus of Elasticity		981355756.8		456271430.9	18.000	-2.558	0.009
								
760, Thin	Peak Load	9	0.013432222	8	0.00632	0.000	-3.464	<0.001
	Modulus of Rupture		1464794.752		670581.0841	0.000	-3.464	<0.001
	Modulus of Elasticity		1962795811		822169413.7	4.000	-3.079	0.001
								
760, Thick	Peak Load	8	0.0404575	12	0.026264167	6.000	-3.242	<0.001
	Modulus of Rupture		1448373.676		843922.4146	0.000	-3.703	<0.001
	Modulus of Elasticity		1180870183		595322917.4	3.000	-3.472	<0.001

The dependent variables of *peak load*, *modulus of rupture*, and *modulus of elasticity* were independently analyzed and are significant. Four tests comparing limestone versus shell were undertaken in each of the three mechanical performance attributes.

## Discussion

Understanding how–in a general sense–the addition of temper to clay produces both production-based and/or performance-based benefits to pottery has long been studied in archaeological research [7, 33, 34, 35, 36, 37]. Less known is the *comparative* performance characteristics of *particular* tempers types relative to other temper types [but see 2, 3, 13]. Here we present a controlled experiment involving several different trials that assesses the performance characteristics of two chemically similar carbonate temper types–limestone and burnt shell–to better understand the potential motivating factors for their selection in prehistory.

Despite their chemical similarity, limestone-tempered samples showed significantly different values in terms of *peak load*, *modulus of rupture*, and *modulus of elasticity*. In terms of withstanding initial fracture (i.e. ceramic “strength”), the assessments of *peak load* and *modulus of rupture* indicated that limestone tempered samples were significantly better than shell tempered samples. Alternately, however, in terms of resisting deformation while under stress from an applied source (i.e. ceramic “toughness”), the shell tempered samples significantly outperformed the limestone ones. In other words, limestone tempered samples were stronger but more brittle (Fig 4), whereas shell tempered samples were weaker but more ductile.

These results have implications for our understanding of prehistoric human selection of temper and the evolution of ceramic technology. Although both carbonate-based tempers are currently thought to offer the same benefits during the initial phase of pottery production, their contrasting post firing properties would have provided distinct benefits in different contexts. Thus, at Late Prehistoric sites like Morrison Village where both limestone and shell tempered pots are found in close association, we can now hypothesize that perhaps pots exhibiting one type of carbonate temper were used in different ways, or for different amounts of time, from pots of another carbonate temper. For example, perhaps limestone tempered pottery was preferred for tasks requiring a vessel with higher initial fracture resistance, such as in transport, whereas shell tempered pottery may have been preferred for tasks requiring a more durable pot that could withstand prolonged pressure after initial fracture. Future assessments of the Morrison Village ceramic assemblage will focus on residue analysis, or other functional indicators, to support or falsify the hypothesis that there may have been a “task-based” differential selection of pottery temper.

## Conclusion

The close association of limestone and burnt shell tempers at the Morrison Village site was the inspiration for the present study. The project goal was to assess the mechanical properties of two calcium carbonate tempers commonly used in prehistoric pottery—limestone and burnt shell. The co-occurrence of two chemically similar tempers generated the research question addressed here: do these two calcium carbonate tempers indeed possess similar mechanical properties related to ceramic strength and toughness? The results of the controlled experimental test do not support the hypothesis that limestone and burnt shell offer the same performance characteristics. These results have implications for our understanding of prehistoric human selection of temper and the evolution of ceramic technology.

We note that the replacement of silicate-based tempers by carbonate-based tempers is not isolated to southern Ohio, nor to North America, but occurs in other regions around the globe [14, 22]. Although we can posit that the initial selective factor for limestone temper over the preceding silicate-based temper was likely due to the role of carbonate temper in vessel formation, why shell temper was ultimately adopted and culturally transmitted, and limestone temper abandoned, remains an important question in light of the current results. Broad-scale assessments of pot function, use-life, and perhaps other variables (e.g. mobility, resource procurement strategies) will help illuminate this question of prehistoric cultural evolution.

## Supporting information

S1 Dataset Instron value generated for each sample: Peak load, modulus of rupture, and modulus of elasticity. (XLSX)
